# Genomic Characterization of Two Novel RCA Phages Reveals New Insights into the Diversity and Evolution of Marine Viruses

**DOI:** 10.1128/Spectrum.01239-21

**Published:** 2021-10-20

**Authors:** Zhiqiang Zhai, Zefeng Zhang, Guiyuan Zhao, Xinxin Liu, Fang Qin, Yanlin Zhao

**Affiliations:** a Fujian Provincial Key Laboratory of Agroecological Processing and Safety Monitoring, College of Life Sciences, Fujian Agriculture and Forestry University, Fuzhou, China; b Key Laboratory of Marine Biotechnology of Fujian Province, Institute of Oceanology, Fujian Agriculture and Forestry University, Fuzhou, China; Huazhong University of Science and Technology

**Keywords:** *Roseobacter* RCA lineage, RCA phages, comparative genomics, phage evolution, functional module

## Abstract

Viruses are the most abundant living entities in marine ecosystems, playing critical roles in altering the structure and function of microbial communities and driving ocean biogeochemistry. Phages that infect *Roseobacter* clade-affiliated (RCA) cluster strains are an important component of marine viral communities. Here, we characterize the genome sequences of two new RCA phages, CRP-9 and CRP-13, which infect RCA strain FZCC0023. Genomic analysis reveals that CRP-9 and CRP-13 represent a novel evolutionary lineage of marine phages. They both have a DNA replication module most similar to those in Cobavirus group phages. In contrast, their morphogenesis and packaging modules are distinct from those in cobaviruses but homologous to those in HMO-2011-type phages. The genomic architecture of CRP-9 and CRP-13 suggests a genomic recombination event between distinct phage groups. Metagenomic data sets were examined for metagenome-assembled viral genomes (MAVGs) with similar recombinant genome architectures. Fifteen CRP-9-type MAVGs were identified from marine viromes. Additionally, 158 MAVGs were identified containing HMO-2011-type morphogenesis and packaging modules with other types of DNA replication genes, providing more evidence that recombination between different phage groups is a major driver of phage evolution. Altogether, this study significantly expands the understanding of diversity and evolution of marine roseophages. Meanwhile, the analysis of these novel RCA phages and MAVGs highlights the critical role of recombination in shaping phage diversity. These phage sequences are valuable resources for inferring the evolutionary connection of distinct phage groups.

**IMPORTANCE** Diversity and evolution of phages that infect the relatively slow-growing but dominant *Roseobacter* lineages are largely unknown. In this study, RCA phages CRP-9 and CRP-13 have been isolated on a *Roseobacter* RCA strain and shown to have a unique genomic architecture, which appears to be the result of a recombination event. CRP-9 and CRP-13 have a DNA replication module most similar to those in Cobavirus group phages and morphogenesis and packaging modules most similar to those in HMO-2011-type phages. HMO-2011-type morphogenesis and packaging modules are found in combination with distinct types of DNA replication genes, suggesting compatibility with various DNA replication modules. Altogether, this study contributes toward a better understanding of marine viral diversity and evolution.

## INTRODUCTION

As the most abundant and ubiquitous biological entities, marine viruses are thought to play critical roles in regulating microbial community structure, affecting the host physiology and evolution, and influencing global biogeochemical cycles (1–3). Marine viruses are morphologically diverse and contain an enormous amount of unstudied genomic diversity (3, 4). In recent years, as next-generation sequencing technologies mature, metagenomic studies have been widely used to explore genetic and functional diversity of marine viruses; they have successfully obtained a large amount of genetic information of double-stranded DNA (dsDNA) metagenome-assembled viral genomes (MAVGs) (5–8). Despite the enormous number of available viral genomes retrieved from metagenomic studies, the majority of MAVGs have limited similarity to phage isolates, and most open reading frames (ORFs) remained unannotated. In addition, the evolutionary history and functional capacity of marine viruses are still poorly understood.

Marine viral communities are mostly composed of phages that infect bacteria (1–3). Phages are known to have pervasively mosaic genomes, resulting from complex evolutionary processes during a long period of time (9–11). Current knowledge of the genetic diversity of phages suggests that genomic evolution of bacteriophages is mainly driven by extensive lateral gene transfer via genetic recombination and fast mutation (12–14). Pairwise genome comparison of over 2,000 phage genomes suggested that phages evolved with low or high gene flux modes, depending on the host, lifestyle, and genomes of phages (15). The most influential theory regarding lateral gene transfer is the theory of modular evolution (16), which proposes that evolution proceeds mainly at the level of functionally interchangeable units (modules). Phage evolution should thus be considered acting on functional modules. In the case of marine bacteriophages, successful (survivable) interchange of genomic modules must entail the continued integrity of the phage function, positive function execution, and functional compatibility (16, 17). The sequences of phage genomes can provide useful information about how the phages have evolved. Therefore, the increasing number of MAVGs is important to understand phage evolution. In recent decades, phages infecting major marine bacteria groups have become a hot spot in marine virology due to the ecological and functional importance of their hosts. Among major bacteria groups, the *Roseobacter* group dominates coastal waters and the polar oceans (18–20). The *Roseobacter* group has diverse pathways for the metabolism of a variety of organic compounds (18–22). Among diverse *Roseobacter* lineages, RCA, CHAB-I-5, SAG-O19, and NAC11-7 dominate, but they are difficult to isolate and cultivate in the lab (23). As the largest lineage of the *Roseobacter* group, the RCA cluster comprises 35% of all planktonic bacteria in the southern ocean and up to 20% to 40% of all roseobacters in temperate and polar oceans (24–26). To date, more than 40 roseophages have been reported (27–30). However, due to difficulties in host cultivation, researchers have only begun to explore the diversity and ecology of phages infecting dominant *Roseobacter* lineages. In 2019, Zhang et al. first reported phages infecting RCA strains (30). These RCA phages are genetically and evolutionarily diverse, belonging to four distinct phage groups (30). Furthermore, phage groups represented by RCA phages have been shown to exhibit wide distribution patterns and high relative abundance compared to other known roseophage groups (30).

Here, we report two novel phages that infect *Roseobacter* RCA strain FZCC0023. Interestingly, the genomes of CRP-9 and CRP-13 seem to contain genomic modules of distinct origins. Genes associated with viral replication are most similar to those in the Cobavirus genomes. Conversely, the morphogenesis and packing-related genes in CRP-9 and CRP-13 are most similar to those in HMO-2011-type phages. Moreover, many similar recombinant MAVGs were identified from marine viromic databases. Our results not only expand the understanding of the roseophages but also have broader implications for understanding the evolution of marine phages.

## RESULTS AND DISCUSSION

### Morphology of CRP-9 and CRP-13.

**FIG 1:**
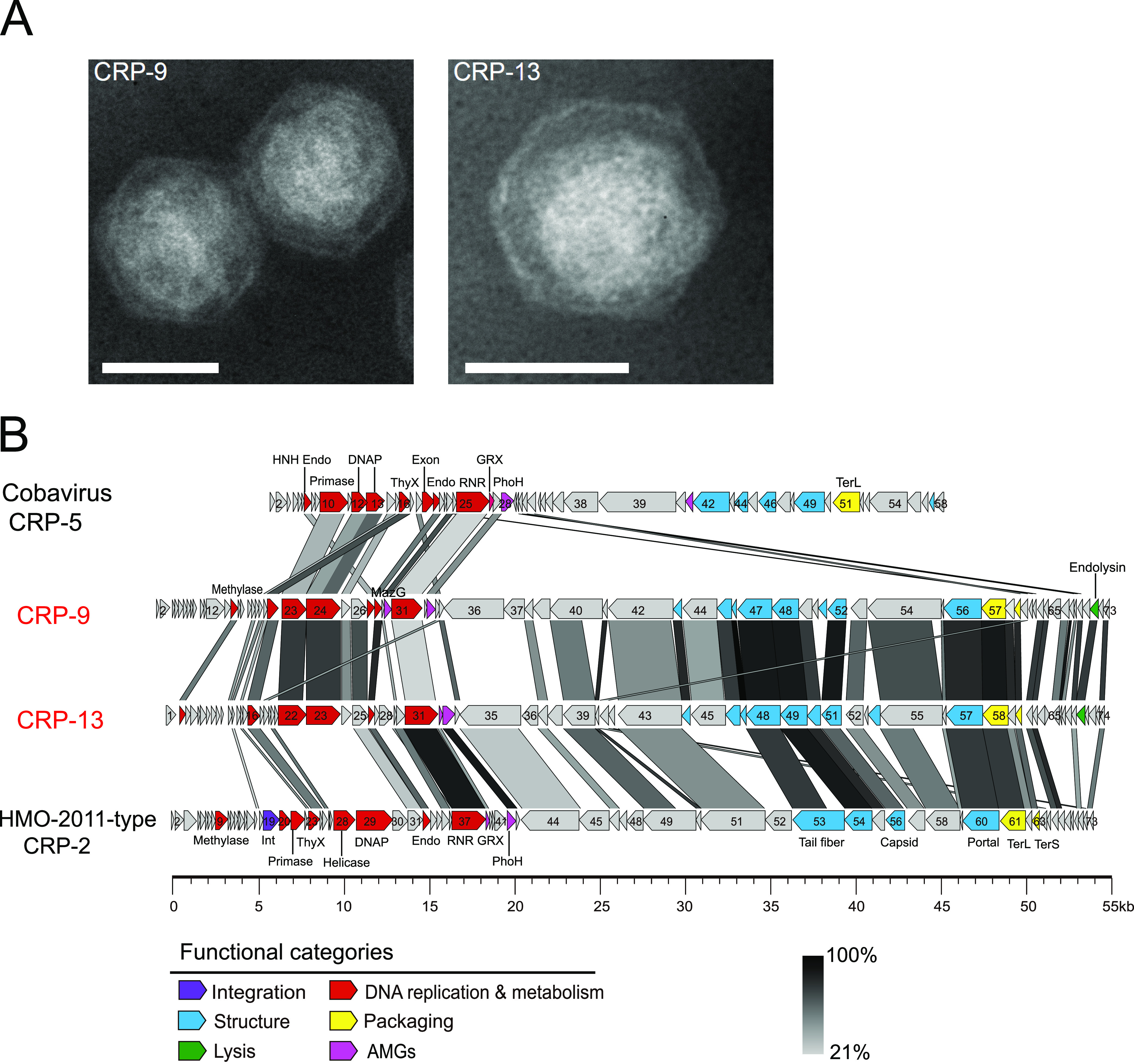
(A) Transmission electron microscopy images of CRP-9 and CRP-13 (scale bars, 50 nm). (B) Genome organization of CRP-9 and CRP-13 showing the combination of Cobavirus-type replication module and HMO-2011-type morphogenesis and packaging modules. ORFs are indicated by a left or right arrow, depending on the direction of transcription. ORFs are color-coded according to putative biological function. Endo, endonuclease; DNAP, DNA polymerase; ThyX, thymidylate synthase; Exon, exonuclease; RNR, ribonucleotide reductase; GRX, glutaredoxin; phoH, phosphate starvation-inducible protein; TerL, terminase large subunit; MazG, MazG nucleotide pyrophosphohydrolase domain protein; Int, integrase; TerS, terminase small subunit.

Negative staining electron microscopy reveals that both CRP-9 and CRP-13 have short tails and isometric heads of similar size in diameter (Fig. 1A), about 70 nm, which are larger than that of the cobaviruses (28, 31) but similar to that of HMO-2011-type RCA phage CRP-1 (30).

### General genomic characteristics of CRP-9 and CRP-13.

The genomic size of CRP-9 is 56,157 bp with 73 predicted ORFs and a tRNA-Arg (TCT) gene (Table 1). CRP-13 is similar to CRP-9 with a genome size of 55,015 bp, encoding 74 ORFs and a tRNA-Arg (TCT) gene. The G+C content of CRP-9 and CRP-13 is 41.6% and 44.2%, respectively, lower than in their host FZCC0023 (53.8%). Overall, CRP-9 and CRP-13 are closely related, sharing approximately half their genes (39 ORFs with 35% to 85% amino acid identity) and have a highly similar genome arrangement (Fig. 1B). Fifty ORFs in CRP-9 and 49 ORFs in CRP-13 have recognizable homologs in NCBI RefSeq; only 19 ORFs in CRP-9 and 17 ORFs in CRP-13 can be assigned to putative biological functions based on sequence homology. The ORFs with known function in CRP-9 and CRP-13 are mostly involved in the essential functions of the phage life cycle, including DNA replication and metabolism, phage morphogenesis, DNA packaging, and cell lysis (Fig. 1B).

**TABLE 1:** General features of RCA phages CRP-9 and CRP-13 in this study

Phage	Source water	Depth	Latitude	Longitude	Date of collection	Genome size (bp)	No. of ORFs	% G+C	GenBank accession no.
CRP-9	Pattaya Beach, Thailand	Surface	12°56′N	100°53′E	March 2018	56,157	73	41.6	MW514246
CRP-11	North Sea	6 m	53°56′N	7°48′E	March 2019	55,015	74	44.2	MW514247

### Modules present in CRP-9 and CRP-13 genomes.

**FIG 2:**
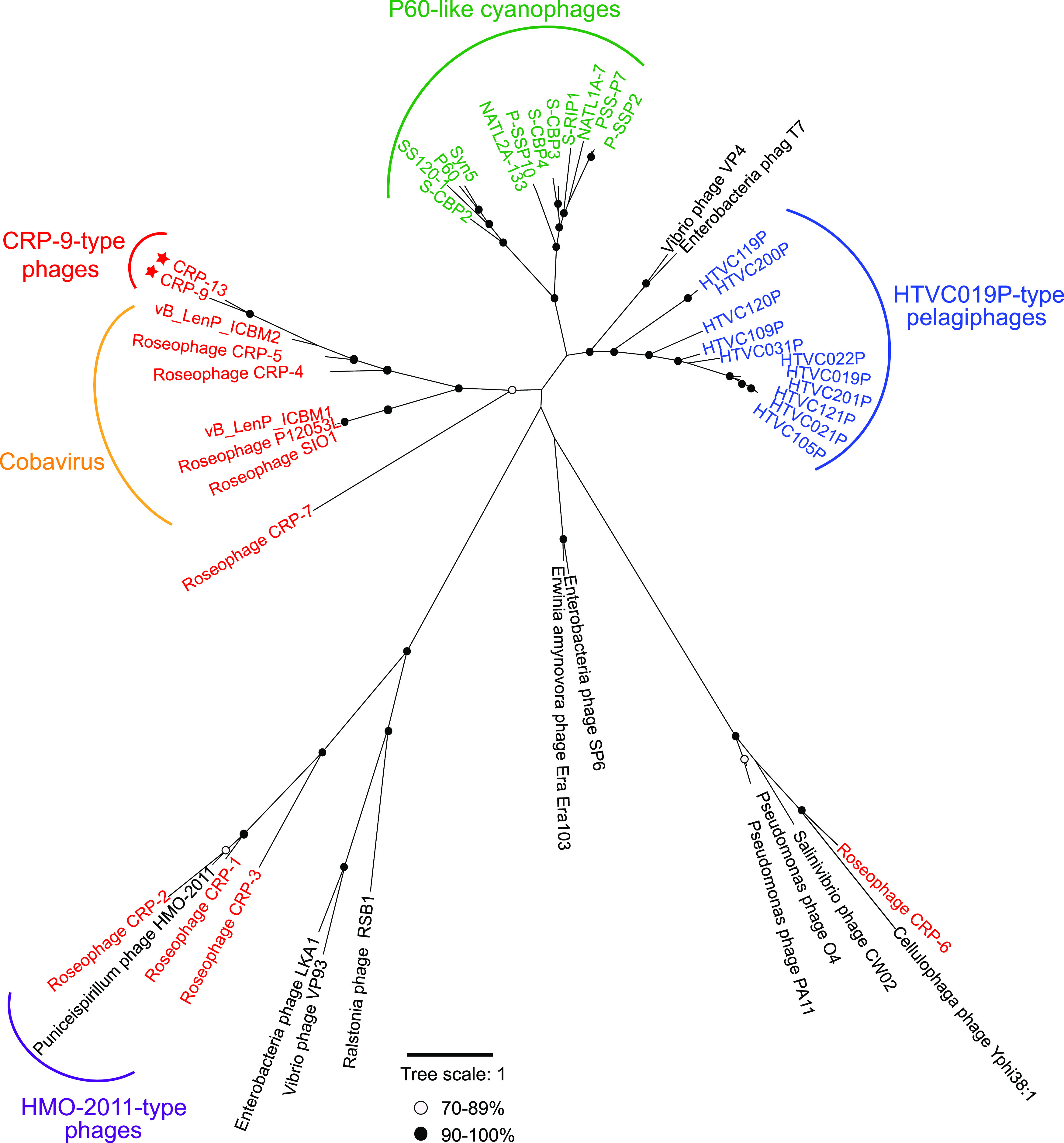
Unrooted maximum-likelihood phylogenetic tree of phage DNA polymerases constructed with conserved polymerase domains. Black and white circles indicate nodes with 90% to 100% and 70% to 89% bootstrap support, respectively. Roseophages, cyanophages, and pelagiphages are shown in red, green, and blue, respectively. Roseophages isolated in this study are marked with red asterisks. Scale bar represents amino acid substitutions per site.

The CRP-9 and CRP-13 genomes each consists of a DNA replication module, a DNA metabolism module, a morphogenesis module, and a DNA packaging module (Fig. 1B). In the DNA replication and metabolism modules, ORFs encoding thymidylate synthase, DNA primase, DNA polymerase, DNA methylase, endonuclease, and ribonucleotide reductase (RNR) were identified. Most genes in this region have similarity with their counterparts in the Cobavirus genomes and are arranged in the same order with cobaviruses (Fig. 1B), suggesting that CRP-9 and CRP-13 share a similar DNA replication strategy with cobaviruses. Among these genes, two DNA replication genes in CRP-9 and CRP-13, DNA primase and DNA polymerase genes, are most similar to those in cobaviruses (28.0% to 60.2% amino acid identity). DNA polymerase phylogeny reveals that CRP-9 and CRP-13 are clustered with cobaviruses, locating on the same branch with CRP-5, CRP-4, and ICBM2 (Fig. 2). Although the DNA replication modules in CRP-9 and CRP-13 show conservation with cobaviruses, another two modules in CRP-9 and CRP-13 display no significant similarity to cobaviruses except for a few homologous ORFs. CRP-9 and CRP-13 genes in the morphogenesis module are most similar to those in HMO-2011-type phage genomes (35% to 99% amino acid identity) (Fig. 1B). The essential proteins encoded by this module include tail structure protein, tail fiber protein, major capsid protein, and portal protein. The terminase large unit and small subunit (TerL and TerS) in their DNA packaging modules are also most similar to those of HMO-2011-type phages (39% to 93% amino acid identity) (Fig. 1B). We also observed that some DNA metabolism genes in CRP-9 and CRP-13 also share identity with those in HMO-2011-type phage genomes.

CRP-9 and CRP-13 can be classified roughly at the genus level based on the phage genus definition criterion (namely, sharing >40% of genes) (32). Here, we refer to this group as the CRP-9-type phage group, characterized as harboring Cobavirus-type DNA replication genes and HMO-2011-type morphogenesis and packaging modules.

The HMO-2011-type and Cobavirus groups are two dominant phage groups in the ocean, exhibiting broad distribution patterns and high relative abundance (28, 30, 33). To date, all cobaviruses were isolated from marine roseobacters (28, 30, 31, 34). The HMO-2011-type group currently has four members; one infects the SAR116 bacterium IMCC1322, and the other three infect RCA roseobacters (30, 33). The genomes of CRP-9 and CRP-13 seem to be the combination of HMO-2011-type and cobaviruses genomes. This novel module combination in the CRP-9 and CRP-13 genomes is of great interest. As the modular theory proposed, functional modules can be shuffled between phage genomes by recombination, resulting in new combinations of modules and thus create potentially novel and viable phages (16). Phage genomic modules can be exchanged with other phages by recombination at specific locations between the modules. It has been suggested that groups of exchanged genes must function together (16). It is currently difficult to track the evolutionary history of this phage type or to identify the boundaries where recombination has occurred, because these phages share limited similarity with other types of phages at the nucleoide level. Lateral gene transfer between phages mainly occurs during coinfection, that is, when more than one phage infects the same host. Coinfection has been identified in some marine bacteria genomic data (35, 36). Such recombination may have occurred between different phages that coexisted within the same host or between a resident prophage and an infecting phage. It is noteworthy that all HMO-2011-type phage isolates encode an integrase gene, implying the ability to establish a lysogenic life cycle (30, 33).

### AMGs identified in CRP-9 and CRP-13.

Auxiliary metabolic genes (AMGs) are a class of phage-encoded metabolic genes speculated to have functions similar to those of their respective host metabolic genes, regulating host metabolism and improving phage adaptability (37, 38). AMGs are usually classified by function into class I and class II. AMGs encoding class I proteins are present in the Kyoto Encyclopedia of Genes and Genomes (KEGG) metabolic pathways, while AMGs encoding class II proteins are not present in KEGG metabolic pathways (38). AMGs are frequently identified in marine roseophage genomes (39). In roseophage genomes, the most prevalent AMGs are those involved in metabolism and nucleotide synthesis (39, 40). This study identified several AMGs in CRP-9 and CRP-13, including the phosphate starvation-inducible protein gene (*phoH*), the nucleoside triphosphate pyrophosphohydrolase gene (*mazG*), and the glutaredoxin gene. The *phoH* genes detected in numerous marine phages have previously been described with diverse function, which includes participating in phospholipid metabolism, RNA modification, and fatty acid beta-oxidation (41, 42). The identification of *phoH* from CRP-9 may suggest that CRP-9 is more successful during infection when the host is undergoing low-phosphate conditions.

The *mazG* gene identified in CRP-9 is a highly conserved gene prevalent in bacterial genomes and has also been identified from many marine phage genomes (30, 33, 39, 43), including all known RCA phages (30). It has been suggested that MazG protein is important for maintaining the starved host metabolism and therefore benefit the propagation of infecting phages (44, 45). In Escherichia coli, MazG has been reported to interfere with the function of the MazEF toxin-antitoxin system by regulating the cellular level of (p)ppGpp (46). However, a recent study showed that a cyanophage MazG has no binding or hydrolysis activity against alarmone (p)ppGpp but has high hydrolytic activity toward dGTP and dCTP, and it was spectated to play a role in hydrolyzing high G+C host genome for phage replication (47). The G+C content of CRP-9 is significantly lower than its host. It is possible that MazG protein in CRP-9 has similar hydrolytic activity to facilitate phage genome replication.

### Prevalence of HMO-2011-type morphogenesis and packaging modules in MAVGs.

**FIG 3:**
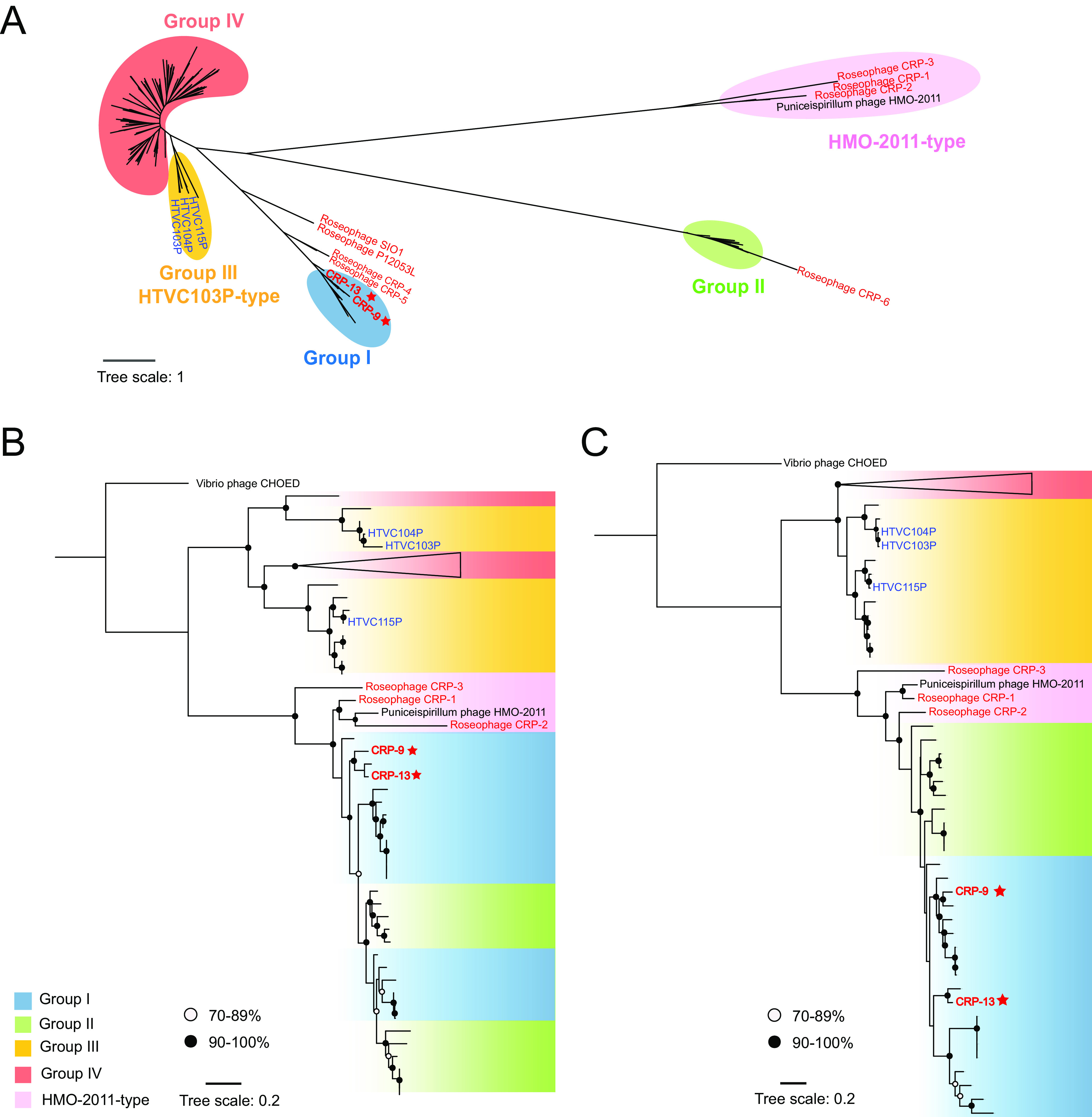
Maximum-likelihood phylogenetic tree of the metagenome-assembled viral genomes (MAVGs). (A) Phylogenetic tree constructed based on DNA polymerase domain sequences. (B) Phylogenetic tree constructed based on major capsid protein sequences. (C) Phylogenetic tree constructed based on TerL sequences. Roseophages and pelagiphages are shown in red and blue, respectively. Roseophages in this study are marked with red stars.

We next sought to investigate the prevalence of the HMO-2011-type morphogenesis and packaging modules in marine MAVGs. A total of 318 MAVGs were retrieved from the Global Ocean Virome 2.0 (GOV 2.0). Genes encoding major capsid, tail protein, portal protein, and terminase in these MAVGs all share high amino acid identity with HMO-2011-type phages, organized in the same order, which suggests close evolutionary relationships. In addition, there are very few insertions and deletions within the HMO-2011-type morphogenesis and packaging modules. Among these 318 MAVGs, 145 contain an HMO-2011-type DNA replication module, thus belonging to the HMO-2011-type phage group. The remaining 173 MAVGs contain DNA replication genes with distinct evolutionary origins (Data Set S1). These results confirm that the HMO-2011-type morphogenesis and packaging modules are present in various phage groups in combination with distinct DNA replication genes. We used the DNA polymerase, capsid, and TerL sequences of these 173 MAVGs for phylogenetic analyses (Fig. 3A, B and C). The capsid and TerL phylogenies also show that all 173 MAVGs cluster with HMO-2011-type phages (Fig. 3B and C).

**FIG 4:**
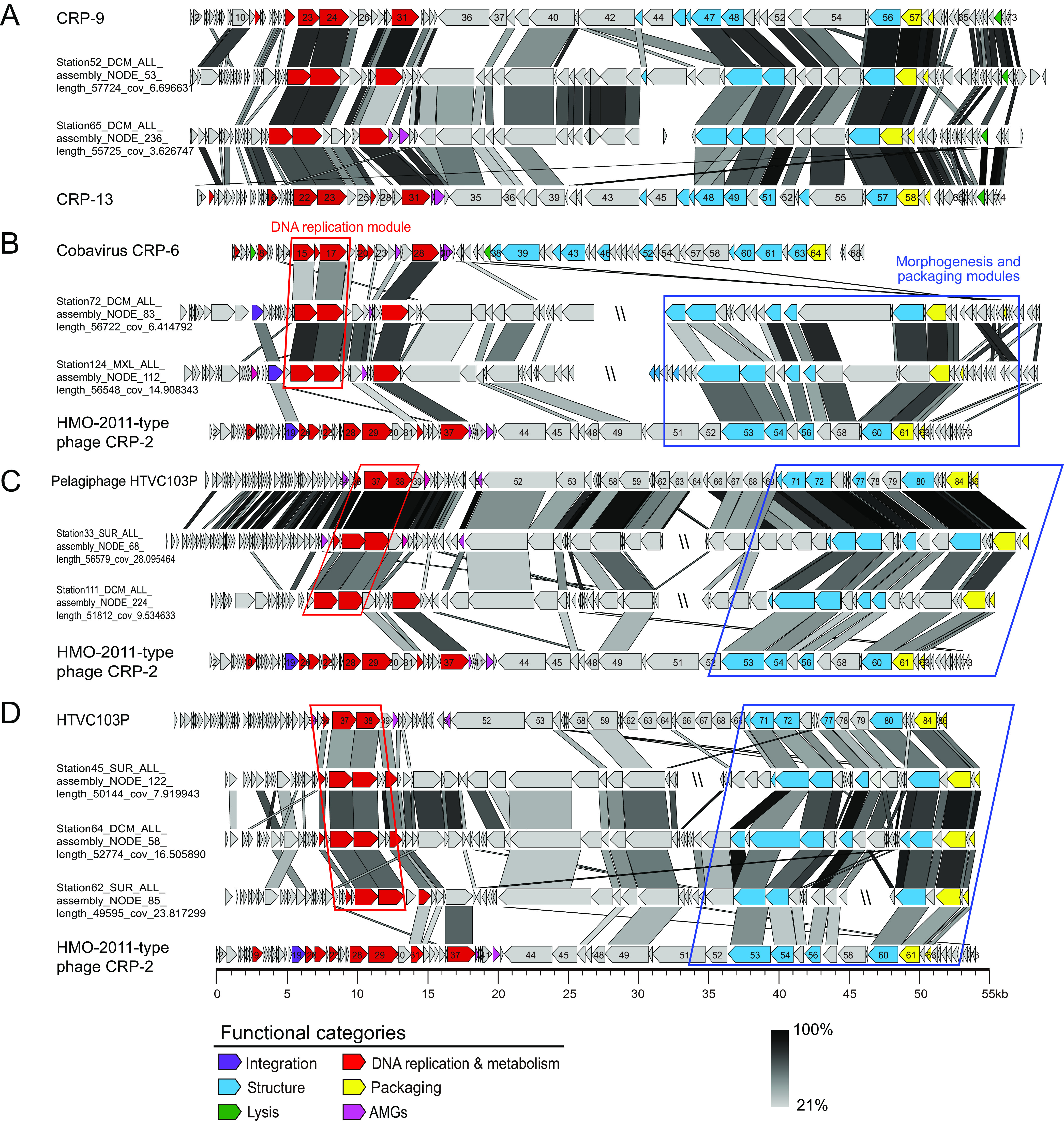
Genomic organization and comparison of representative metagenome-assembled viral genomes (MAVGs). (A) Group I, CRP-9-type MAVGs. (B) Group II, MAVGs containing a CRP-6 DNA replication module. (C) Group III, HTVC103P-type MAVGs. (D) Group IV, unclassified MAVGs. ORFs are color-coded according to their putative biological function. The color of the shading connecting homologous genes indicates the level of amino acid identities between the genes. Similar DNA replication modules are boxed in red, and similar morphogenesis and packaging modules are boxed in blue.

A conserved DNA polymerase domain (PF00476)-based phylogenetic analysis separated the 173 MAVGs into four major groups (I to IV), distinct from the HMO-2011-type group (Fig. 3A). Among these 173 MAVGs, 15 are associated with CRP-9 and CRP-13 in the DNA polymerase tree (Fig. 3A, group I). Genomic comparison shows that all these 15 MAVGs are largely syntenic to CRP-9 and CRP-13 throughout the whole-genome region (Fig. 4A). Therefore, all 15 MAVGs belong to the CRP-9-type phage group.

Twelve MAVGs are shown to cluster with RCA phage CRP-6 in the DNA polymerase tree (Fig. 3A, group II). CRP-6 represents a novel phage type, sharing limited homology with other known phage isolates (30). Genomic comparison shows that these 12 MAVGs and CRP-6 have a very similar DNA replication module but are distinct in morphogenesis and packaging modules (Fig. 4B). All 12 MAVGs contain a set of DNA replication genes most similar to CRP-6, including DNA primase, DNA polymerase, and a few other associated genes of unknown function (Fig. 4B). We noticed that the majority, 10 of these 12 MAVGs, contain an integrase gene homologous to that in HMO-2011-type phages (36.5% to 42.7% amino acid identity). In contrast, an integrase gene was not identified in other groups. In HMO-2011-type phages, integrase gene is located upstream of DNA replication genes (30, 33). In other MAVGs, the integrase genes may have been lost during recombination, or their ancestor did not contain the integrase gene.

The remaining 146 MAVGs, we found, are more closely related to HTVC103P-type pelagiphages in the DNA polymerase tree and can be further divided into two groups (Fig. 3C, group III and IV). As in CRP-9 and CRP-13, a remarkable feature of the HTVC103P-type group is that these pelagiphages all harbor a set of morphogenesis and packaging genes similar to their counterparts in HMO-2011-type genomes (33). Genomic comparison reveals remarkable synteny among group III MAVGs and HTVC103P-type pelagiphages (Fig. 4C). They share 33.3% to 97.7% of their ORFs with HTVC103P-type pelagiphages and have conserved DNA replication, morphogenesis, and packaging modules with HTVC103P-type pelagiphages. Thus, they all fall within the HTVC103P-type phage group and may infect SAR11 bacteria. In addition, the G+C content of group III MAVGs ranged from 30.8% to 34.0%, similar to the G+C content of all known pelagiphages. There is an obvious but more distant relationship between group IV MAVGs and HTVC103P-type pelagiphages (Fig. 3C; Fig. 4D). Compared with group III genomes, they share a relatively lower percentage of their ORFs (20.9 to 65.9%) with HTVC103P-type pelagiphages and have a wider range of G+C content (31.9 to 44.4%). Due to the lack of cultured representatives in this group, the hosts of group IV remain to be further explored.

The analysis of these MAVGs reveals that similar HMO-2011-type morphogenesis and packaging modules can exist in combination with various DNA replication modules. In each case, we found the DNA polymerase gene location closely associated with the helicase gene, and each DNA polymerase-helicase combination was exclusive to each group. In phage genomes, DNA polymerase reacts with DNA helicase in the DNA replication process (48). Clearly, a particular DNA polymerase-helicase combination is vital for a functional DNA replication module. Due to the intimate interactions of DNA replication genes, some phages that undergo DNA replication module replacement survive to infect a host.

The novel module combination of CRP-9, CRP-13, and these MAVGs supports the modular nature of phage genomes. Similar observations were previously reported for several phage genomes (10, 12, 49–54). Collectively, these results indicate that genetic recombination plays a major role in the diversification of phage genomes, producing novel combinations of genomic modules. Many phage types have arisen from recombination between two phage ancestors.

**FIG 5:**
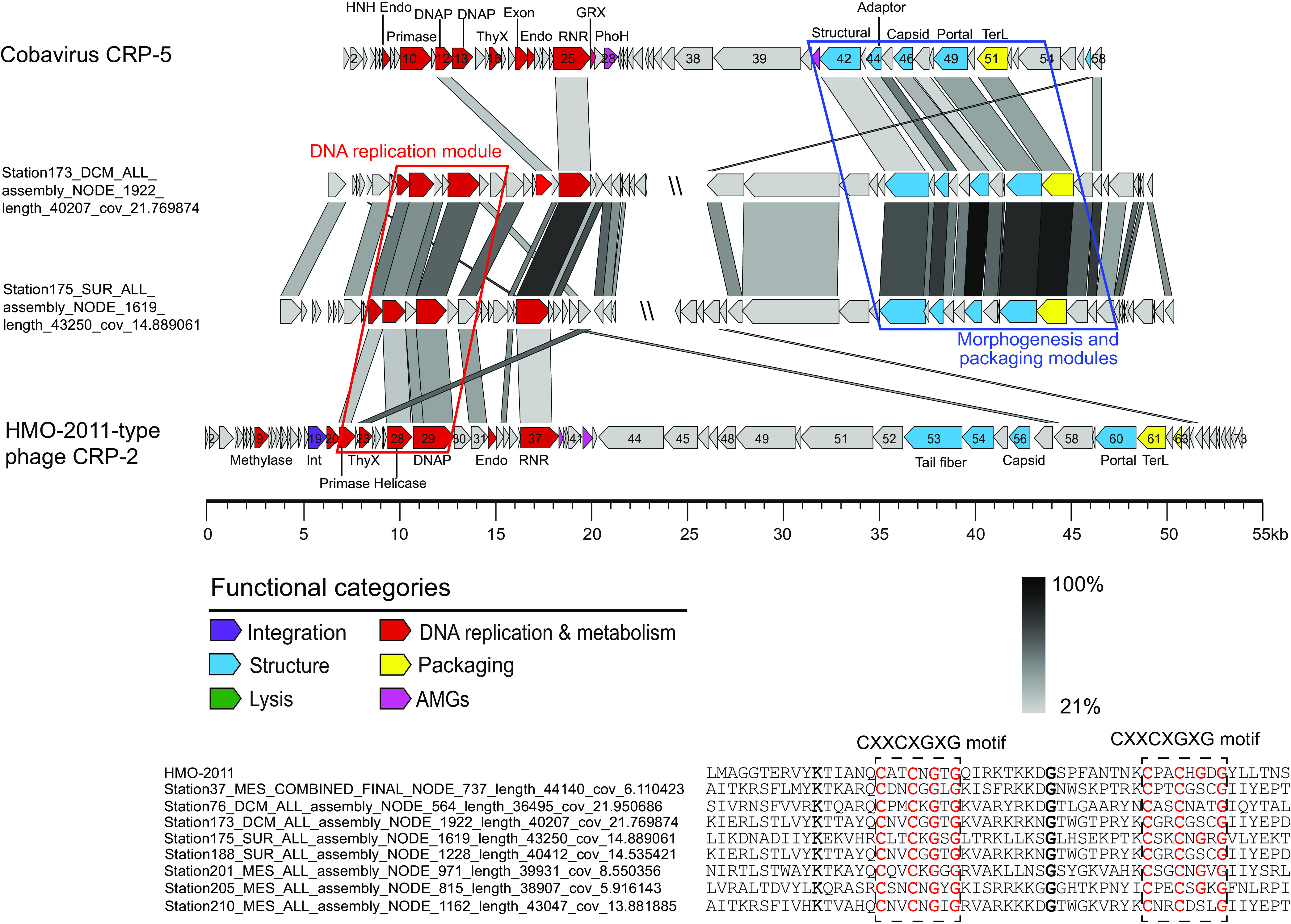
Genome organization of MAVGs showing the combination of HMO-2011-type replication module and Cobavirus-type morphogenesis and packaging module. ORFs are color-coded based on their putative biological function. The color of the shading connecting homologous genes indicates the level of amino acid identities between the genes. Similar DNA replication modules are boxed in red, and similar morphogenesis and packaging modules are boxed in blue. Two CXXCXGXG motifs are boxed with dotted lines; conserved residues in them are marked by red color.

Phage genomes generally have a higher frequency of recombination to mutation (55). The recombination rate is also highly variable across the genome regions. The horizontal exchange of sequences among phage genomes is thought to be rampant between phage genomes and randomly distributed over the genomes (9). Only a minor fraction of sequence exchange, without interrupting module function, can produce functional and competitive recombinants. Based on our observation, it can be speculated that DNA replication modules were transferred between phage genomes, which resulted in new combinations of functional modules and production of new phage types. The identification of these MAVGs suggests that HMO-2011-type morphogenesis and packaging modules are functionally compatible with various DNA replication modules. Existing phages with these types of combinations have survived natural selection. We searched for MAVGs containing the HMO-2011-type DNA replication module and the Cobavirus-like morphogenesis and packaging modules. A total of 8 corresponding MAVGs were successfully retrieved from the GOV 2.0 database (Fig. 5). These 8 MAVGs all contain an HMO-2011-type DNA replication module with DNA polymerases having a conserved DnaJ domain (box in Fig. 5) (33). The discovery of this MAVG genotype further supports the idea that recombination plays a critical role in generating novel phage genotypes.

Due to the prominent role of recombination in the diversification of bacteriophage genomes, the assessment of phylogenetic relationships among different phage groups is challenging. Many phage genome sections display different evolutionary histories. In these cases, classification of these phages is challenging, phylogenetic analysis based on a single hallmark gene can be problematic, and network analysis based on gene content may require caution.

In some studies, some “hybrid” phage gene modules have a high level of nucleotide identity with other types of phage gene modules through whole-genome nucleotide comparisons, suggesting that the exchange events occurred relatively recently (10, 56). However, in this study, it was difficult to reconstruct the modular recombination history of these phages; the precise boundaries of these “modules” have remained elusive using current genomic data due to CRP-9-type genomes lacking nucleotide-level similarity with other types of phages.

### Conclusion.

In this study, a novel type of marine RCA phages has been identified, providing more insights into the diversity and evolution of marine RCA phages. They both possess a Cobavirus-type DNA replication module, suggesting that they use the same DNA replication strategy with cobaviruses. However, their morphogenesis and packaging modules share common ancestry with HMO-2011-type phages. This is the first case of Cobavirus-type DNA replication genes being found outside the Cobavirus group. These two RCA phages and recombinant forms of MAVGs confirm that the recombination of modules among phages is an important evolutionary process that shapes the structure of marine phages. The global marine phage population may harbor a much higher diversity of genomic architectures than has been previously recognized.

## MATERIALS AND METHODS

### Host strain and growth condition.

*Roseobacter* RCA strain FZCC0023 was isolated from coastal water of Pingtan Island, China in 2017 by dilution to extinction method (30). The detailed phylogenetic information on FZCC0023 has been described in an earlier study (30). FZCC0023 was grown at 23°C in modified natural seawater-based medium amended with 1 μM FeCl_3_, excess vitamins, 1 mM NH_4_Cl, 100 μM KH_2_PO_4_, and mixed carbon source (57).

### Source waters and isolation of CRP-9 and CRP-13.

A seawater sample was collected from the coast of Pattaya Beach, Thailand (latitude, 12°56′N; longitude, 100°53′E) in March 2018. The other seawater sample was collected from the German Bight, North Sea (latitude, 53°56′N; longitude, 7°48′E) in March 2019, by the RV Heincke during the cruise HE526 (58) (Table 1). Water samples were filtered through a 0.1-μm syringe filter (Pall Gelman Laboratory, USA) and stored at 4°C until use. The phages were isolated according to the procedure described in previous reports (30). Briefly, 0.1 μm filtered seawater was added to exponentially growing FZCC0023 cultures. The cultures were incubated at 23°C until cell lysis was detected by using a Guava easyCyte flow cytometer (Merck Millipore, Billerica, MA). The presence of phage particles was also confirmed by epifluorescence microscopy. To obtain the pure phage clone, the extinction dilution procedure (59) was repeated three times. The purity of the RCA phages was examined by genome sequencing.

### Transmission electron microscopy.

CRP-9 and CRP-13 lysates were filtered through 0.1-μm-pore-size filters and then concentrated by Amicon Ultra centrifugal filters (30 kDa; Merck Millipore). Next, CRP-9 and CRP-13 were further concentrated by an ultracentrifuge (Beckman Coulter, USA) at 50,000 × *g* for 2 h. The concentrated phage samples were adsorbed onto a copper grid in the dark and stained with 2% uranyl acetate for 2 min. A Hitachi transmission electron microscope was used to observe the samples at 80 kV.

### Phage DNA extraction.

The preparation and concentration of phage lysates were carried out as described by Zhang et al. (30) Briefly, 250 ml of host culture was infected with each phage at a phage-to-host ratio of approximately 3:1. After host lysis, the phage lysates were filtered through 0.1-μm-pore-size filters and then further concentrated by Amicon Ultra centrifugal filters (30 kDa; Merck Millipore). Phage DNA was extracted using a formamide treatment, phenol-chloroform extraction method (60). Subsequently, the phage genomes were sequenced with paired-end sequencing by Mega Genomics (Beijing, China) using the HiSeq 2500 sequencing system (Illumina). The Illumina raw reads obtained were quality controlled, adaptor trimmed, and assembled using CLC Genomic Workbench 11.0.1 software (Qiagen, Hilden, Germany) with default settings.

### Genome annotation.

The open reading frames (ORFs) of CRP-9 and CRP-13 were predicted by GeneMark (61). The translated ORFS were analyzed by BLASTP (E value < 1E-3, coverage ≥ 50%, amino acid identity ≥ 25%) query against the NCBI nonredundant protein sequences (nr) and NCBI reference proteins (refseq_protein) databases. Proteins were also used to search the PFAM (https://pfam.xfam.org) (62) and HHpred (https://toolkit.tuebingen.mpg.de/tools/hhpred) databases (63) to identify protein families and distant protein homologs, respectively. tRNA gene identification was performed with tRNAscan-SE (64).

### Retrieval of MAVGs.

A total of 515,588 MAVGs from the following metagenomic data sets were downloaded for analysis: (i) the Med-DCM fosmid library (5), (ii) the Global Ocean Viromes (7), and (iii) the Global Ocean Virome 2.0 (GOV 2.0) (8). The following search strategy was used to retrieve the MAVGs. First, HMO-2011-type capsid and TerL sequences (including sequences from HMO-2011-type phages, CRP-9, CRP-13, and HTVC103P-type pelagiphages) were searched against genomes of MAVGs using TBLASTN (E value < 1E-3, coverage ≥ 80%), resulting in 1,242 MAVGs that contain both HMO-2011-type capsid and TerL genes. After this initial search, a search was conducted to identify DNA polymerase genes in the MAVGs. Among the 1,242 MAVGs, 318 MAVGs had a DNA polymerase domain (PF00476) and were retained for further analysis. Next, 145 MAVGs containing an HMO-2011-type DNA polymerase were excluded from further analysis. The remaining 173 MAVGs all contain the HMO-2011-type morphogenesis and packing modules, but their DNA replication modules are distinct from HMO-2011-type phages (Data set S1). All 173 MAVGs were recovered from GOV 2.0, and no MAVGs were obtained from the Med-DCM fosmids or GOV data sets. The above processes were applied to retrieve MAVGs containing HMO-2011-type morphogenesis and packing modules plus other types of DNA replication modules.

We also searched for MAVGs containing the HMO-2011-type replication genes and the Cobavirus-type morphogenesis and packing modules. In brief, Cobavirus-like capsid and TerL sequences (including P12053L, CRP-4, CRP-5, vB_LenP_ICBM1, and vB_LenP_ICBM2) were searched against genomes of MAVGs by BLASTP (E value < 1E-3, coverage ≥ 80%), resulting in 438 MAVGs. A DNA polymerase domain was identified from 144 of these 438 MAVGs. Next, HMO-2011-type DNA polymerase sequences were searched against 144 MAVGs genomes by TBLASTN (E value < 1E-3, coverage ≥ 70%). A conserved DnaJ domain containing two repeats of the CXXCXGXG motif was used as the criterion to identify HMO-2011-type DNA polymerases (33).

### Phylogenetic analysis.

Multiple alignments were computed using MUSCLE software (65) and edited with Gblocks (66). ModelFinder (67) was used to evaluate optimal amino acid substitution models for alignments. Phylogenetic trees were constructed using the IQTREE v2.0.3 with 1,000 ultrafast bootstrap replicates (68).

### Data availability.

The genome sequences of CRP-9 and CRP-13 have been deposited in the GenBank database under accession numbers MW514246 and MW514247.

## References

[1] Fuhrman JA. 1999. Marine viruses and their biogeochemical and ecological effects. Nature 399:541–548. doi:10.1038/21119. 10376593

[2] Wommack KE, Colwell RR. 2000. Virioplankton: viruses in aquatic ecosystems. Microbiol Mol Biol Rev 64:69–114. doi:10.1128/MMBR.64.1.69-114.2000. 10704475 PMC98987

[3] Suttle CA. 2007. Marine viruses–major players in the global ecosystem. Nat Rev Microbiol 5:801–812. doi:10.1038/nrmicro1750. 17853907

[4] Dion MB, Oechslin F, Moineau S. 2020. Phage diversity, genomics and phylogeny. Nat Rev Microbiol 18:125–138. doi:10.1038/s41579-019-0311-5. 32015529

[5] Mizuno CM, Rodriguez-Valera F, Kimes NE, Ghai R. 2013. Expanding the marine virosphere using metagenomics. PLoS Genet 9:e1003987. doi:10.1371/journal.pgen.1003987. 24348267 PMC3861242

[6] Brum JR, Ignacio-Espinoza JC, Roux S, Doulcier G, Acinas SG, Alberti A, Chaffron S, Cruaud C, de Vargas C, Gasol JM, Gorsky G, Gregory AC, Guidi L, Hingamp P, Iudicone D, Not F, Ogata H, Pesant S, Poulos BT, Schwenck SM, Speich S, Dimier C, Kandels-Lewis S, Picheral M, Searson S, Bork P, Bowler C, Sunagawa S, Wincker P, Karsenti E, Sullivan MB, Tara Oceans Coordinators. 2015. Patterns and ecological drivers of ocean viral communities. Science 348:1261498. doi:10.1126/science.1261498. 25999515

[7] Roux S, Brum JR, Dutilh BE, Sunagawa S, Duhaime MB, Loy A, Poulos BT, Solonenko N, Lara E, Poulain J, Pesant S, Kandels-Lewis S, Dimier C, Picheral M, Searson S, Cruaud C, Alberti A, Duarte CM, Gasol JM, Vaqué D, Bork P, Acinas SG, Wincker P, Sullivan MB, Tara Oceans Coordinators. 2016. Ecogenomics and potential biogeochemical impacts of globally abundant ocean viruses. Nature 537:689–693. doi:10.1038/nature19366. 27654921

[8] Gregory AC, Zayed AA, Conceição-Neto N, Temperton B, Bolduc B, Alberti A, Ardyna M, Arkhipova K, Carmichael M, Cruaud C, Dimier C, Domínguez-Huerta G, Ferland J, Kandels S, Liu Y, Marec C, Pesant S, Picheral M, Pisarev S, Poulain J, Tremblay JÉ, Vik D, Babin M, Bowler C, Culley AI, de Vargas C, Dutilh BE, Iudicone D, Karp-Boss L, Roux S, Sunagawa S, Wincker P, Sullivan MB, Tara Oceans Coordinators. 2019. Marine DNA viral macro- and microdiversity from pole to pole. Cell 177:1109–1123. doi:10.1016/j.cell.2019.03.040. 31031001 PMC6525058

[9] Hendrix RW, Smith MC, Burns RN, Ford ME, Hatfull GF. 1999. Evolutionary relationships among diverse bacteriophages and prophages: all the world's a phage. Proc Natl Acad Sci USA 96:2192–2197. doi:10.1073/pnas.96.5.2192. 10051617 PMC26759

[10] Hendrix RW. 2008. Phage evolution, p 177–194. *In* Abedon ST (ed), Bacteriophage ecology. Cambridge University Press, Cambridge, UK.

[11] Hatfull GF, Hendrix RW. 2011. Bacteriophages and their genomes. Curr Opin Virol 1:298–303. doi:10.1016/j.coviro.2011.06.009. 22034588 PMC3199584

[12] Pedulla ML, Ford ME, Houtz JM, Karthikeyan T, Wadsworth C, Lewis JA, Jacobs-Sera D, Falbo J, Gross J, Pannunzio NR, Brucker W, Kumar V, Kandasamy J, Keenan L, Bardarov S, Kriakov J, Lawrence JG, Jacobs WR, Jr., Hendrix RW, Hatfull GF. 2003. Origins of highly mosaic mycobacteriophage genomes. Cell 113:171–182. doi:10.1016/s0092-8674(03)00233-2. 12705866

[13] Lucchini S, Desiere F, Brüssow H. 1999. Comparative genomics of *Streptococcus thermophilus* phage species supports a modular evolution theory. J Virol 73:8647–8656. doi:10.1128/JVI.73.10.8647-8656.1999. 10482618 PMC112885

[14] Anne K, Horst N, Huang KD, Hoeppner MP, Heller KJ, Franz CMAP, Dagan T. 2018. Rates of mutation and recombination in *Siphoviridae* phage genome evolution over three decades. Mol Biol Evol 35:1147–1159. doi:10.1093/molbev/msy027. 29688542 PMC5913663

[15] Mavrich TN, Hatfull GF. 2017. Bacteriophage evolution differs by host, lifestyle and genome. Nat Microbiol 2:17112. doi:10.1038/nmicrobiol.2017.112. 28692019 PMC5540316

[16] Botstein D. 1980. A theory of modular evolution for bacteriophages. Ann N Y Acad Sci 354:484–490. doi:10.1111/j.1749-6632.1980.tb27987.x. 6452848

[17] Veesler D, Cambillau C. 2011. A common evolutionary origin for tailed-bacteriophage functional modules and bacterial machineries. Microbiol Mol Biol Rev 75:423–433. doi:10.1128/MMBR.00014-11. 21885679 PMC3165541

[18] Buchan A, González JM, Moran MA. 2005. Overview of the marine *Roseobacter* lineage. Appl Environ Microbiol 71:5665–5677. doi:10.1128/AEM.71.10.5665-5677.2005. 16204474 PMC1265941

[19] Wagner-Döbler I, Biebl H. 2006. Environmental biology of the marine *Roseobacter* lineage. Annu Rev Microbiol 60:255–280. doi:10.1146/annurev.micro.60.080805.142115. 16719716

[20] Brinkhoff T, Giebel HA, Simon M. 2008. Diversity, ecology, and genomics of the *Roseobacter* clade: a short overview. Arch Microbiol 189:531–539. doi:10.1007/s00203-008-0353-y. 18253713

[21] Moran MA, Belas R, Schell MA, González JM, Sun F, Sun S, Binder BJ, Edmonds J, Ye W, Orcutt B, Howard EC, Meile C, Palefsky W, Goesmann A, Ren Q, Paulsen I, Ulrich LE, Thompson LS, Saunders E, Buchan A. 2007. Ecological genomics of marine roseobacters. Appl Environ Microbiol 73:4559–4569. doi:10.1128/AEM.02580-06. 17526795 PMC1932822

[22] Newton RJ, Griffin LE, Bowles KM, Meile C, Gifford S, Givens CE, Howard EC, King E, Oakley CA, Reisch CR, Rinta-Kanto JM, Sharma S, Sun S, Varaljay V, Vila-Costa M, Westrich JR, Moran MA. 2010. Genome characteristics of a generalist marine bacterial lineage. ISME J 4:784–798. doi:10.1038/ismej.2009.150. 20072162

[23] Zhang Y, Sun Y, Jiao N, Stepanauskas R, Luo H. 2016. Ecological genomics of the uncultivated marine *Roseobacter* lineage CHAB-I-5. Appl Environ Microbiol 82:2100–2111. doi:10.1128/AEM.03678-15. 26826224 PMC4807517

[24] Selje N, Simon M, Brinkhoff T. 2004. A newly discovered *Roseobacter* cluster in temperate and polar oceans. Nature 427:445–448. doi:10.1038/nature02272. 14749832

[25] Giebel HA, Brinkhoff T, Zwisler W, Selje N, Simon M. 2009. Distribution of *Roseobacter* RCA and SAR11 lineages and distinct bacterial communities from the subtropics to the Southern Ocean. Environ Microbiol 11:2164–2178. doi:10.1111/j.1462-2920.2009.01942.x. 19689707

[26] Giebel HA, Kalhoefer D, Lemke A, Thole S, Gahl-Janssen R, Simon M, Brinkhoff T. 2011. Distribution of *Roseobacter* RCA and SAR11 lineages in the North Sea and characteristics of an abundant RCA isolate. ISME J 5:8–19. doi:10.1038/ismej.2010.87. 20596072 PMC3105675

[27] Zhan Y, Chen F. 2019. Bacteriophages that infect marine roseobacters: genomics and ecology. Environ Microbiol 21:1885–1895. doi:10.1111/1462-2920.14504. 30556267

[28] Bischoff V, Bunk B, Meier-Kolthoff JP, Spröer C, Poehlein A, Dogs M, Nguyen M, Petersen J, Daniel R, Overmann J, Göker M, Simon M, Brinkhoff T, Moraru C. 2019. Cobaviruses - a new globally distributed phage group infecting *Rhodobacteraceae* in marine ecosystems. ISME J 13:1404–1421. doi:10.1038/s41396-019-0362-7. 30718806 PMC6775973

[29] Cai L, Ma R, Chen H, Yang Y, Jiao N, Zhang R. 2019. A newly isolated roseophage represents a distinct member of *Siphoviridae* family. Virol J 16:128. doi:10.1186/s12985-019-1241-6. 31694663 PMC6836515

[30] Zhang Z, Chen F, Chu X, Zhang H, Luo H, Qin F, Zhai Z, Yang M, Sun J, Zhao Y. 2019. Diverse, abundant, and novel viruses infecting the marine *Roseobacter* RCA lineage. mSystems 4:e00494-19. doi:10.1128/mSystems.00494-19. 31848303 PMC6918029

[31] Rohwer F, Segall A, Steward G, Seguritan V, Breitbart M, Wolven F, Farooq AF. 2000. The complete genomic sequence of the marine phage *Roseophage* SIO1 shares homology with nonmarine phages. Limnol Oceanogr 45:408–418. doi:10.4319/lo.2000.45.2.0408.

[32] Lavigne R, Seto D, Mahadevan P, Ackermann HW, Kropinski AM. 2008. Unifying classical and molecular taxonomic classification: analysis of the *Podoviridae* using BLASTP-based tools. Res Microbiol 159:406–414. doi:10.1016/j.resmic.2008.03.005. 18555669

[33] Kang I, Oh HM, Kang D, Cho JC. 2013. Genome of a SAR116 bacteriophage shows the prevalence of this phage type in the oceans. Proc Natl Acad Sci USA 110:12343–12348. doi:10.1073/pnas.1219930110. 23798439 PMC3725092

[34] Kang I, Jang H, Oh HM, Cho JC. 2012. Complete genome sequence of *Celeribacter* bacteriophage P12053L. J Virol 86:8339–8340. doi:10.1128/JVI.01153-12. 22787270 PMC3421633

[35] Roux S, Hawley AK, Torres Beltran M, Scofield M, Schwientek P, Stepanauskas R, Woyke T, Hallam SJ, Sullivan MB. 2014. Ecology and evolution of viruses infecting uncultivated SUP05 bacteria as revealed by single-cell- and meta-genomics. Elife 3:e03125. doi:10.7554/eLife.03125. 25171894 PMC4164917

[36] Roux S, Hallam SJ, Woyke T, Sullivan MB, 2015. Viral dark matter and virus-host interactions resolved from publicly available microbial genomes. Oceanography 20:e08490. doi:10.7554/eLife.08490. PMC4533152 26200428

[37] Breitbart M. 2012. Marine viruses: truth or dare. Annu Rev Mar Sci 4:425–448. doi:10.1146/annurev-marine-120709-142805. 22457982

[38] Hurwitz BL, U'Ren JM. 2016. Viral metabolic reprogramming in marine ecosystems. Curr Opin Microbiol 31:161–168. doi:10.1016/j.mib.2016.04.002. 27088500

[39] Huang X, Jiao N, Zhang R. 2021. The genomic content and context of auxiliary metabolic genes in roseophages. Environ Microbiol 23:3743–3757. doi:10.1111/1462-2920.15412. 33511765

[40] Yang Y, Cai L, Ma R, Xu Y, Tong Y, Huang Y, Jiao N, Zhang R. 2017. A novel roseosiphophage isolated from the oligotrophic South China Sea. Viruses 9:109. doi:10.3390/v9050109. PMC5454422 28505134

[41] Goldsmith DB, Crosti G, Dwivedi B, McDaniel LD, Varsani A, Suttle CA, Weinbauer MG, Sandaa RA, Breitbart M. 2011. Development of phoH as a novel signature gene for assessing marine phage diversity. Appl Environ Microbiol 77:7730–7739. doi:10.1128/AEM.05531-11. 21926220 PMC3209181

[42] Goldsmith DB, Parsons RJ, Beyene D, Salamon P, Breitbart M. 2015. Deep sequencing of the viral phoH gene reveals temporal variation, depth-specific composition, and persistent dominance of the same viral phoH genes in the Sargasso Sea. PeerJ 3:e997. doi:10.7717/peerj.997. 26157645 PMC4476143

[43] Crummett LT, Puxty RJ, Weihe C, Marston MF, Martiny JBH. 2016. The genomic content and context of auxiliary metabolic genes in marine cyanomyoviruses. Virology 499:219–229. doi:10.1016/j.virol.2016.09.016. 27693926

[44] Bryan MJ, Burroughs NJ, Spence EM, Clokie MR, Mann NH, Bryan SJ. 2008. Evidence for the intense exchange of MazG in marine cyanophages by horizontal gene transfer. PLoS One 3:e2048. doi:10.1371/journal.pone.0002048. 18431505 PMC2297514

[45] Duhaime MB, Wichels A, Waldmann J, Teeling H, Glöckner FO. 2011. Ecogenomics and genome landscapes of marine *Pseudoalteromonas* phage H105/1. ISME J 5:107–121. doi:10.1038/ismej.2010.94. 20613791 PMC3105678

[46] Gross M, Marianovsky I, Glaser G. 2006. MazG–a regulator of programmed cell death in *Escherichia coli*. Mol Microbiol 59:590–601. doi:10.1111/j.1365-2958.2005.04956.x. 16390452

[47] Rihtman B, Bowman-Grahl S, Millard A, Corrigan RM, Clokie MRJ, Scanlan DJ. 2019. Cyanophage MazG is a pyrophosphohydrolase but unable to hydrolyse magic spot nucleotides. Environ Microbiol Rep 11:448–455. doi:10.1111/1758-2229.12741. 30809954 PMC6850273

[48] Spacciapoli P, Nossal NG. 1994. Interaction of DNA polymerase and DNA helicase within the bacteriophage T4 DNA replication complex. Leading strand synthesis by the T4 DNA polymerase mutant A737V (tsL141) requires the T4 gene 59 helicase assembly protein. J Biol Chem 269:447–455. doi:10.1016/S0021-9258(17)42371-4. 8276834

[49] Jäckel C, Hammerl JA, Reetz J, Kropinski AM, Hertwig S. 2015. *Campylobacter* group II phage CP21 is the prototype of a new subgroup revealing a distinct modular genome organization and host specificity. BMC Genomics 16:629. doi:10.1186/s12864-015-1837-1. 26296758 PMC4546147

[50] McDonnell B, Mahony J, Neve H, Hanemaaijer L, Noben JP, Kouwen T, van Sinderen D. 2016. Identification and analysis of a novel group of bacteriophages infecting the lactic acid bacterium *Streptococcus thermophilus*. Appl Environ Microbiol 82:5153–5165. doi:10.1128/AEM.00835-16. 27316953 PMC4988201

[51] Zhan Y, Huang S, Voget S, Simon M, Chen F. 2016. A novel roseobacter phage possesses features of podoviruses, siphoviruses, prophages and gene transfer agents. Sci Rep 6:30372. doi:10.1038/srep30372. 27460944 PMC4961962

[52] Johnson MC, Sena-Velez M, Washburn BK, Platt GN, Lu S, Brewer TE, Lynn JS, Stroupe ME, Jones KM. 2017. Structure, proteome and genome of Sinorhizobium meliloti phage ΦM5: a virus with LUZ24-like morphology and a highly mosaic genome. J Struct Biol 200:343–359. doi:10.1016/j.jsb.2017.08.005. 28842338

[53] Šulčius S, Šimoliūnas E, Alzbutas G, Gasiūnas G, Jauniškis V, Kuznecova J, Miettinen S, Nilsson E, Meškys R, Roine E, Paškauskas R, Holmfeldt K. 2019. Genomic characterization of cyanophage vB_AphaS-CL131 infecting filamentous diazotrophic cyanobacterium *Aphanizomenon flos-aquae* reveals novel insights into virus-bacterium interactions. Appl Environ Microbiol 85:e01311-18. doi:10.1128/AEM.01311-18. 30367000 PMC6293099

[54] Philippe C, Levesque S, Dion MB, Tremblay DM, Horvath P, Lüth N, Cambillau C, Franz C, Neve H, Fremaux C, Heller KJ, Moineau S. 2020. Novel genus of phages infecting S*treptococcus thermophilus*: genomic and morphological characterization. Appl Environ Microbiol 86:e00227-20. doi:10.1128/AEM.00227-20. 32303549 PMC7301855

[55] Stedman KM. 2018. Viral recombination: ecology, evolution, and pathogenesis. Viruses 10:358. doi:10.3390/v10070358. PMC6070879 29986376

[56] Ford ME, Sarkis GJ, Belanger AE, Hendrix RW, Hatfull GF. 1998. Genome structure of mycobacteriophage D29: implications for phage evolution. J Mol Biol 279:143–164. doi:10.1006/jmbi.1997.1610. 9636706

[57] Cho JC, Giovannoni SJ. 2004. Cultivation and growth characteristics of a diverse group of oligotrophic marine *Gammaproteobacteria*. Appl Environ Microbiol 70:432–440. doi:10.1128/AEM.70.1.432-440.2004. 14711672 PMC321273

[58] Alfred-Wegener-Institut Helmholtz-Zentrum für Polar- und Meeresforschung. 2017. Research vessel HEINCKE operated by the Alfred-Wegener-Institute. Journal of Large-Scale Research Facilities 3:A120. doi:10.17815/jlsrf-3-164.

[59] Nagasaki K, Gunnar B. 2010. Isolation of viruses infecting photosynthetic and nonphotosynthetic protists, p 92–101. *In* Wilhelm SW, Weinbauer MG, Suttle CA (ed), Manual of aquatic viral ecology. Association for the Sciences of Limnology and Oceanography, Waco, TX.

[60] Sambrook J, Fritsch EF, Maniatis T. 2001. Molecular cloning: a laboratory manual, 3rd ed. Cold Spring Harbor Laboratory Press, Cold Spring Harbor, NY.

[61] Borodovsky M, McIninch J. 1993. GENMARK: parallel gene recognition for both DNA strands. Comput Chem 17:123–133. doi:10.1016/0097-8485(93)85004-V.

[62] Finn RD, Bateman A, Clements J, Coggill P, Eberhardt RY, Eddy SR, Heger A, Hetherington K, Holm L, Mistry J, Sonnhammer EL, Tate J, Punta M. 2014. Pfam: the protein families database. Nucleic Acids Res 42:D222–D230. doi:10.1093/nar/gkt1223. 24288371 PMC3965110

[63] Söding J, Biegert A, Lupas AN. 2005. The HHpred interactive server for protein homology detection and structure prediction. Nucleic Acids Res 33:W244–W248. doi:10.1093/nar/gki408. 15980461 PMC1160169

[64] Lowe TM, Eddy SR. 1997. tRNAscan-SE: a program for improved detection of transfer RNA genes in genomic sequence. Nucleic Acids Res 25:955–964. doi:10.1093/nar/25.5.955. 9023104 PMC146525

[65] Edgar RC. 2004. MUSCLE: multiple sequence alignment with high accuracy and high throughput. Nucleic Acids Res 32:1792–1797. doi:10.1093/nar/gkh340. 15034147 PMC390337

[66] Castresana J. 2000. Selection of conserved blocks from multiple alignments for their use in phylogenetic analysis. Mol qBiol Evol 17:540–552. doi:10.1093/oxfordjournals.molbev.a026334. 10742046

[67] Kalyaanamoorthy S, Minh BQ, Wong TKF, von Haeseler A, Jermiin LS. 2017. ModelFinder: fast model selection for accurate phylogenetic estimates. Nat Methods 14:587–589. doi:10.1038/nmeth.4285. 28481363 PMC5453245

[68] Nguyen LT, Schmidt HA, von Haeseler A, Minh BQ. 2015. IQ-TREE: a fast and effective stochastic algorithm for estimating maximum-likelihood phylogenies. Mol Biol Evol 32:268–274. doi:10.1093/molbev/msu300. 25371430 PMC4271533

